# Personalized management of glucokinase-related monogenic diabetes (GCK-MODY) during pregnancy: a case report

**DOI:** 10.3389/fendo.2026.1859312

**Published:** 2026-05-28

**Authors:** Ana Filipa Bolas, João Oliveira Torres, Paula Bogalho, José Silva-Nunes

**Affiliations:** 1 Endocrinology, Diabetes and Metabolism Department, Hospital de Curry Cabral, Unidade Local de Saúde São José, Lisbon, Portugal; 2 NOVA Medical School, Faculdade de Ciências Médicas, Universidade Nova de Lisboa, Lisbon, Portugal; 3 Centro Clínico Académico de Lisboa, Lisbon, Portugal; 4 Faculdade de Medicina, Universidade de Lisboa, Lisbon, Portugal

**Keywords:** case report, diabetes, GCK, MODY, monogenic diabetes, pregnancy

## Abstract

**Background:**

Management of glucokinase-related monogenic diabetes (GCK-MODY) during pregnancy can be challenging.

**Case presentation:**

We present the case report of a 33-year-old woman, diagnosed with diabetes since the age of 9. She had no micro or macrovascular complications, and HbA1c ranged between 6.5% and 6.8%, without pharmacological treatment. Family history revealed multiple relatives with the diagnosis of diabetes, including a maternal cousin with confirmed GCK-MODY. The patient underwent genetic testing that identified the variant NM_000162.5:c.829_830insCGG p.(Leu276_Val277insAla), heterozygous for the GCK gene, classified as of uncertain significance. During pregnancy, the couple chose not to undergo invasive fetal testing. Given the presumptive diagnosis of GCK-MODY and since fetal genotype was not determined, the pregnancy was managed under the assumption of a potentially unaffected fetus. During follow-up, combined with nutritional counseling and promotion of regular exercise, the patient required initiation of basal insulin therapy. Then, to optimize metabolic control, continuous glucose monitoring (CGM) was implemented, and insulin therapy was progressively intensified to a basal-bolus regimen, ultimately reaching eight daily bolus administrations of rapid-acting insulin. Adequate glycemic control and appropriate fetal growth were achieved, and she delivered a male newborn at 36 weeks and 2 days, with 2970g and APGAR score of 9 at 1, 5 and 10 minutes. Genetic testing of the mother and sister was requested during the patient’s pregnancy, but the results became available only after delivery. These confirmed the presence of the same variant in both relatives, supporting the diagnosis of GCK-MODY.

**Conclusion:**

This case highlights the complexity of managing presumptive GCK-MODY during pregnancy, in the setting of a variant of uncertain significance and absent fetal genotyping. It emphasizes the importance of CGM-guided intensive insulin therapy and the need for a multidisciplinary and individualized approach based on shared decision-making.

## Introduction

Maturity-onset diabetes of the young (MODY) is the most common form of monogenic diabetes, accounting for 1-5% of all cases of diabetes. Heterozygous inactivating variants in the glucokinase (GCK) gene result in GCK-MODY. GCK is a key glucose-sensing enzyme in pancreatic beta-cells that is responsible for glucose-mediated insulin secretion. Heterozygous inactivating mutations cause a mild, lifelong hyperglycemia by raising the glucose threshold for insulin secretion, typically resulting in fasting plasma glucose levels of 99–144 mg/dL (5.5–8.0 mmol/L). Most patients are asymptomatic, microvascular and macrovascular complications are rare, and pharmacological treatment is often not required (1–3). However, recognition of GCK-MODY during pregnancy is crucial, as the treatment approach may differ significantly from that used in gestational diabetes. Fetal growth is primarily determined by the inheritance of the maternal GCK mutation by the fetus, and maternal glycemic control is relevant only when the fetus does not inherit the GCK mutation (4, 5).

We present the case of a woman managed as probable GCK-MODY during pregnancy, considering the presence of a variant of uncertain significance, highlighting the challenges associated with diagnosis, decision-making in the absence of fetal genotyping, and the need for individualized insulin therapy guided by CGM.

## Case presentation

A 33-year-old woman, G0P0, BMI 19.2 kg/m^2^, had been diagnosed with diabetes since she was 9 years old. She was initially diagnosed with type 2 diabetes and started treatment with oral antihyperglycemic agents, which she discontinued due to lack of effectiveness. Later, at the age of 32, she was referred to the Endocrinology Consultation to further clarify the diagnosis. She had no microvascular or macrovascular complications diagnosed, had never experienced an acute metabolic decompensation, HbA1c ranged between 6.5% and 6.8%, and she had never required insulin therapy. Family history revealed diabetes in multiple relatives across successive generations, including the patient’s younger sister, mother, maternal grandmother, maternal uncle, maternal aunt, and a maternal cousin, the latter with a confirmed diagnosis of GCK-MODY. The patient underwent genetic testing with next-generation sequencing that identified the variant NM_000162.5:c.829_830insCGG p.(Leu276_Val277insAla), heterozygous for the GCK gene, classified as of uncertain significance. Despite this result, the overall clinical phenotype supported the presumptive diagnosis of GCK-MODY, and the patient was monitored accordingly.

During pregnancy, the woman was followed by Endocrinology from the 7th week of gestation until delivery, with evaluations every one to two weeks. The couple was referred to genetic counseling, but they chose not to undergo invasive fetal testing, given the risks associated with the procedure. The initial glycemic profile revealed fasting and postprandial hyperglycemia. Given the diagnostic uncertainty and the absence of fetal genotyping, glycemic targets were individualized throughout pregnancy. At 7 weeks of gestation, therapy with insulin glargine 5 units/day was initiated to achieve an initial fasting glycemic target of <110 mg/dL (<6.1 mmol/L), to avoid overtreatment and unnecessary maternal hypoglycemia. At 8 weeks of gestation, due to persistent hyperglycemia, insulin glargine was increased to 12 units/day, and the fasting glycemic target was decreased to <95 mg/dL (<5.3 mmol/L), in line with gestational diabetes recommendations, given the concern for potential fetal overexposure to maternal hyperglycemia. Following this insulin increment, the patient began to report symptoms consistent with hypoglycemia, including tremor, sweating and a sensation of hunger, predominantly in the fasting state, despite maintaining normal glycemic measurements, with fasting values ranging from 102–144 mg/dL (5.7–8.0 mmol/L) and 1-hour postprandial values of 147–203 mg/dL (8.2–11.3 mmol/L). At 11 weeks of gestation, insulin glargine was decreased to 6 units/day, continuous glucose monitoring (CGM) was initiated and insulin lispro was introduced before the main meals, with redefinition of the glycemic targets: fasting glucose to 100–120 mg/dL (5.6–6.7 mmol/L), and postprandial glucose to <140 mg/dL (<7.8 mmol/L). CGM data, including fasting glucose trends and postprandial excursions, guided further therapeutic adjustments. Daily insulin requirements, CGM parameters, and fetal parameters, according to gestational age, are presented in **Table 1**. Throughout the pregnancy, insulin therapy was progressively intensified, reaching eight daily administrations of insulin lispro, alongside basal insulin therapy and nutritional and exercise interventions. During this period, the time below range, defined as <100 mg/dL (<5.6 mmol/L), to avoid symptoms of hypoglycemia, varied between 0% and 3%. Glycemic control improved progressively, and by the 35th week of gestation the patient was receiving insulin glargine 6 units/day and insulin lispro 29.5 units/day, achieving a time in range (TIR), defined as 100–140 mg/dL (5.6–7.8 mmol/L), of 95%. At that point, time below range was 3%, with just 1% of the time falling below 70 mg/dL (3.9 mmol/L).

**Table 1:** Daily insulin requirements, CGM parameters, and fetal parameters during pregnancy.

Weeks of pregnancy	Basal insulin (units)	Prandial insulin (units)	Daily dose of insulin (units)	Time in range (%)	Time above range (%)	Time below range (%)	EFW (g) (P) AC (cm) HC/AC ratio
11 – 13	6	6	12	81	17	2	
13 – 15	6	6.5	12.5	81	18	1	
15 – 17	6	8.5	14.5	85	15	0	
17 – 19	6	11.5	17.5	90	9	1	216 (P32) 12.8 1.12
19 – 21	6	11.5	17.5	90	10	0	
21 – 23	6	15	21	87	13	0	487 (P48) 17.4 1.1
23 – 25	6	17	23	82	18	0	
25 – 27	6	19.5	25.5	84	15	1	
27 – 29	6	20.5	26.5	91	9	0	1288 (P46) 24.6 1.05
29 – 31	6	24	30	95	5	0	
31 – 33	6	24	30	88	9	3	
33 – 35	6	29.5	35.5	95	2	3	2362 (P44) 30.1 1.02

EFW, Estimated fetal weight; P, percentile, AC, abdominal circumference, HC, head circumference.

Obstetric follow-up was uneventful throughout the pregnancy, with normal and frequent ultrasounds with adequate fetal growth (44th-48th percentile), abdominal circumference and head circumference/abdominal circumference ratio, as well as fetal echocardiograms without structural abnormalities.

She was hospitalized at 36 weeks and 2 days of gestation due to spontaneous membrane rupture. She underwent a forceps-assisted delivery due to non-reassuring fetal status and dystocia of progression. A male newborn of 2970g was born, with an APGAR score of 9 at 1, 5 and 10 minutes, without complications in the postpartum period. After delivery, insulin therapy was discontinued. She maintained follow-up at the Diabetes Consultation, with an optimal metabolic control without any pharmacological intervention.

Furthermore, to support variant interpretation, genetic testing of the mother and sister was requested during the patient’s pregnancy. However, the results became available only after delivery and confirmed the presence of the same variant in both relatives, supporting the diagnosis of GCK-MODY.

## Discussion

This case highlights the challenges of managing presumptive GCK-MODY during pregnancy, in the presence of a variant of uncertain significance and when fetal genotyping is unavailable. In this context, decisions regarding glycemic targets, insulin initiation, and treatment intensification had to be individualized according to maternal metabolic evolution, CGM findings, and serial fetal ultrasound assessment.

MODY is frequently underrecognized and misclassified, resulting in delayed diagnosis and incorrect treatment (1, 3, 6). In particular, GCK-MODY is often misclassified as type 2 diabetes due to its clinical presentation, leading many patients to receive unnecessary pharmacological therapy prior to genetic diagnosis, despite treatment generally not being required outside the context of pregnancy (1–3). Diagnostic confirmation can be particularly challenging as many mutations have not yet been identified or are not considered pathogenic. According to the Human Gene Mutation Database^®^, more than one thousand pathogenic mutations have been described in the GCK gene, with most being missense variants affecting the structural and functional integrity of glucokinase (7, 8). To our knowledge, the variant NM_000162.5:c.829_830insCGG p.(Leu276_Val277insAla) has not been reported in major public databases, nor has it been described in the scientific literature to date. This in-frame insertion occurs within a conserved region of the enzyme, where several pathogenic variants have previously been identified (9). In our patient, recognition of probable GCK-MODY occurred only 23 years after the initial clinical presentation of diabetes. Diagnostic interpretation was further limited by the identification of a variant classified as of uncertain significance in the databases used in our genetics laboratory, and additional familial segregation data confirming the same variant only became available after the child’s birth.

Regarding the management of GCK-MODY during pregnancy, there are specific challenges. Treatment decision must consider the fetal genotype, since inappropriate glucose control can adversely affect fetal development. If the fetus inherits the maternal GCK mutation, achieving strict glycemic targets with insulin may suppress fetal insulin secretion, as the normal maternal glucose levels are insufficient to stimulate the defective beta-cells, potentially leading to reduced fetal growth. Therefore, in these cases, pharmacological treatment is generally not required. Conversely, if the fetus does not carry the mutation, exposure to maternal hyperglycemia can induce fetal malformations and stimulate excessive fetal insulin production, which increases the risk of macrosomia, caesarean section and neonatal hypoglycemia. However, genetic testing is not always available. Thus, current evidence supports a conservative approach to managing GCK-MODY during pregnancy, prioritizing frequent fetal growth monitoring by ultrasound and using glycemic safety thresholds to initiate insulin (2, 4, 10–13). Nevertheless, this approach has significant limitations during early pregnancy (2, 12, 13). Although it is possible to assess abdominal circumference after the 14th week of gestation, its accuracy and clinical usefulness for guiding therapeutic decisions is only consolidated in the end of the second trimester of pregnancy (14). This initial window of time creates a period of clinical uncertainty that extends for more than half of the pregnancy, generating significant stress for both the couple and the healthcare professionals involved. On the one hand, there is the concern of not treating a condition that could lead to fetal macrosomia; on the other hand, in fetuses carrying the mutation, there is the risk of instituting unnecessary therapy, increasing the risk of maternal hypoglycemia and potential intrauterine growth restriction (10, 13). Additionally, this uncertainty is further aggravated by the fact that the period of fetal organogenesis, when the teratogenic risk of hyperglycemia is greatest, occurs precisely during the first trimester, when ultrasound evaluation is not accurately informative (15). For this reason, the development of non-invasive prenatal genetic testing represents a significant advance in the management of GCK-MODY during pregnancy. By enabling the analysis of circulating free fetal DNA in maternal plasma, fetal genotype can be determined from 12 weeks of gestation onwards with high accuracy (100% sensitivity; 96% specificity) (12), surpassing the accuracy of conventional ultrasound (10, 12, 16). The early availability of this result will allow for personalized management from the beginning of pregnancy, optimizing disease management and reducing its emotional impact.

Continuous glucose monitoring (CGM) has also emerged as a valuable tool in the management of GCK-MODY during pregnancy, particularly in early gestational weeks, allowing for more accurate assessment of glycemic patterns and real-time therapeutic adjustments. Recent data suggest that CGM metrics may guide the need for insulin treatment early in pregnancy when fetal genotype is unknown, by assessing the time in range during pregnancy (defined as 63–140 mg/dL [3.5–7.8 mmol/L]) and the glucose management index (GMI). However, it is important to emphasize that women with GCK-MODY often do not achieve pregnancy targets due to the fasting hyperglycemia inherent to the disease, and the therapeutic goal should be the upper limit of the TIR rather than euglycemia, to avoid growth restriction in fetuses carrying the mutation (17). In our case, the identified GCK variant remained classified as a variant of uncertain significance, and the couple declined invasive fetal testing. Consequently, therapeutic decisions had to rely on maternal metabolic phenotype, CGM-guided evaluation, and serial ultrasound assessments, from the moment it was possible to perform this assessment. CGM played a central role throughout pregnancy, allowing close assessment of glucose trends, which guided insulin titration and therapeutic escalation.

Additionally, management is further challenged by maternal symptoms of hypoglycemia at normal glucose levels, making the traditional glycemic targets for gestational diabetes difficult to achieve (2, 10). Recommended strategies include frequent blood glucose monitoring, individualized adjustment of antihyperglycemic therapy only when there is evidence of accelerated fetal growth, and close monitoring by a multidisciplinary team including Endocrinology, Obstetrics, Genetics, and Nutrition specialists (4, 11). Indeed, this patient initially presented with symptoms of hypoglycemia, despite having blood glucose levels within the normal range. This made conventional gestational diabetes glycemic targets difficult to tolerate and highlighted the limitations of applying standardized targets in pregnancies managed under genetic uncertainty. Following individualized therapeutic adjustment, redefinition of glycemic targets, and specialized nutritional intervention, hypoglycemic episodes became rare, without worsening of glycemic control.

Maternal and neonatal outcomes in GCK-MODY are generally favorable when managed appropriately (4, 11). The incidence of congenital malformations is low (2.4%), both in mutation carriers and non-carrier fetuses (5). Regarding the mode of delivery, literature shows that the cesarean section rate is often higher among women in whom insulin therapy was initiated during pregnancy (11). In our case report, maternal and neonatal outcomes were favorable, highlighting the importance of a multidisciplinary approach, rigorous monitoring, and shared decision-making throughout pregnancy.

## Conclusion

This case report highlights the challenges of managing presumptive GCK-MODY during pregnancy and emphasizes the potential role of CGM-guided intensive insulin therapy to ensure optimized metabolic control, especially when fetal genotype is unknown. Although the management of GCK-MODY during pregnancy currently relies primarily on ultrasound assessment, in the future, routine implementation of non-invasive prenatal genetic testing will enable a more personalized approach, ultimately improving maternal-fetal outcomes.

## Data Availability

The original contributions presented in the study are included in the article/**Supplementary Material**. Further inquiries can be directed to the corresponding author.
